# Transcriptome analysis of symptomatic and recovered leaves of geminivirus-infected pepper (*Capsicum annuum*)

**DOI:** 10.1186/1743-422X-9-295

**Published:** 2012-11-27

**Authors:** Elsa Góngora-Castillo, Enrique Ibarra-Laclette, Diana L Trejo-Saavedra, Rafael F Rivera-Bustamante

**Affiliations:** 1 Departamento de Ingeniería Genética, Centro de Investigación y de Estudios Avanzados del I.P.N (Cinvestav)-Unidad Irapuato, Km 9.6 Libramiento Norte, Carretera Irapuato-León, Irapuato, Gto., 36821, México; 2 Laboratorio Nacional de Genómica para la Biodiversidad (Langebio), Cinvestav-Irapuato, Km 9.6 Libramiento Norte, Carretera Irapuato-León, Irapuato, Gto., 36821, México

**Keywords:** Differential expression, Geminiviruses, *Pepper golden mosaic virus*, Plant defense, Recovery

## Abstract

**Background:**

Geminiviruses are a large and important family of plant viruses that infect a wide range of crops throughout the world. The *Begomovirus* genus contains species that are transmitted by whiteflies and are distributed worldwide causing disease on an array of horticultural crops. Symptom remission, in which newly developed leaves of systemically infected plants exhibit a reduction in symptom severity (recovery), has been observed on pepper (*Capsicum annuum*) plants infected with *Pepper golden mosaic virus* (PepGMV). Previous studies have shown that transcriptional and post-transcriptional gene silencing mechanisms are involved in the reduction of viral nucleic acid concentration in recovered tissue. In this study, we employed deep transcriptome sequencing methods to assess transcriptional variation in healthy (mock), symptomatic, and recovered pepper leaves following PepGMV infection.

**Results:**

Differential expression analyses of the pepper leaf transcriptome from symptomatic and recovered stages revealed a total of 309 differentially expressed genes between healthy (mock) and symptomatic or recovered tissues. Computational prediction of differential expression was validated using quantitative reverse-transcription PCR confirming the robustness of our bioinformatic methods. Within the set of differentially expressed genes associated with the recovery process were genes involved in defense responses including pathogenesis-related proteins, reactive oxygen species, systemic acquired resistance, jasmonic acid biosynthesis, and ethylene signaling. No major differences were found when compared the differentially expressed genes in symptomatic and recovered tissues. On the other hand, a set of genes with novel roles in defense responses was identified including genes involved in histone modification. This latter result suggested that post-transcriptional and transcriptional gene silencing may be one of the major mechanisms involved in the recovery process. Genes orthologous to the *C. annuum* proteins involved in the pepper-PepGMV recovery response were identified in both *Solanum lycopersicum* and *Solanum tuberosum* suggesting conservation of components of the viral recovery response in the Solanaceae.

**Conclusion:**

These data provide a valuable source of information for improving our understanding of the underlying molecular mechanisms by which pepper leaves become symptomless following infection with geminiviruses. The identification of orthologs for the majority of genes differentially expressed in recovered tissues in two major solanaceous crop species provides the basis for future comparative analyses of the viral recovery process across related taxa.

## Background

Geminiviruses are a large and important family of plant viruses that infect a wide variety of crops around the world. The family *Geminiviridae* is divided into four genera (*Mastrevirus, Curtovirus, Begomovirus, Topocuvirus*) based on genome organization (mono- or bipartite), insect vector (whiteflies, leafhoppers, treehoppers), and host range (monocotyledonous or dicotyledonous plant species). Geminiviral genomes are composed of circular, single-stranded DNA (ssDNA) molecules encapsidated in twin icosahedral virions for which the family is named [1,2]. The ssDNA viral genomes are transcribed, replicated, and encapsidated in the nuclei of infected cells. Geminiviruses also traffic within and between host cells moving systemically throughout the infected plant and are dependent on host machinery for both replication and movement [3,4].

Species of the genus *Begomovirus* are transmitted by whiteflies (*Bemisia tabaci* Genn.) and distributed worldwide causing diseases in horticultural crops such as tomato and pepper [5-7]. Crop losses of up to 100% have been reported for geminivirus diseases [8]. *Pepper golden mosaic virus* (PepGMV) is one of the most important and widely distributed *Begomovirus* throughout Mexico and infects several *Solanaceae* crops including pepper (*Capsicum annuum*), tomato (*Solanum lycopersicum*), and tomatillo (*Physalis ixocarpa*) [8-10]. The bipartite genome of PepGMV encodes six proteins. The DNA-A component encodes proteins involved in replication (Rep and REn), trans-activation (TrAP), and the capsid protein (CP) whereas viral DNA-B encodes proteins related to movement (NSP and MP) [10]. PepGMV infection results in bright yellow mosaic symptoms on leaves that is associated with twisting and distortion of leaves and fruits, stunted plants, and reduced yield [11].

**Figure 1:**
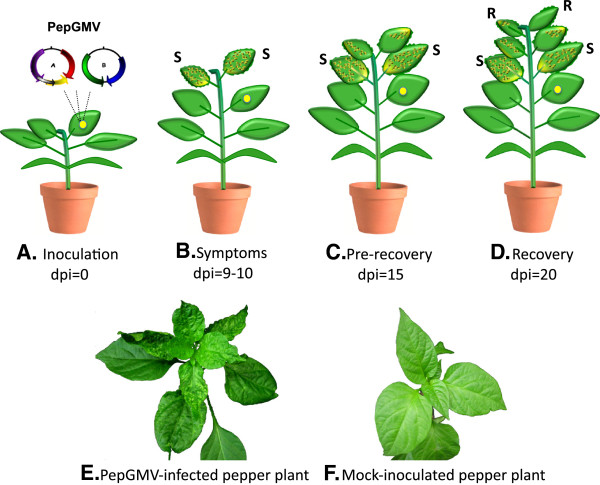
**Schematic of the recovery process.** Depiction of the infection, symptom, pre-recovery, and recovery stages in the PepGMV-pepper system. **A**) Inoculation: Pepper plants at the four leaf stage were inoculated with dimeric clones of PepGMV using biolistics [12]. **B**) Symptoms: New emerging leaves show typical PepGMV infection symptoms at 9–10 dpi (symptomatic stage). **C**) Pre-recovery: At 15 dpi, newly emergent leaves have decreased severity of the symptoms (pre-recovered stage). **D**) Recovery: Leaves emerge with a recovered phenotype or a significant decrease in the symptom severity around 20 dpi (recovered stage). **E**) PepGMV-infected pepper plant at 20 dpi, showing the recovery process. **F**) Healthy (Mock-inoculated) pepper plant.

In studies under controlled conditions, symptom remission has been observed on PepGMV-infected pepper plants [12]. Thus, in the pepper-PepGMV recovery system, pepper plants show severe symptoms around 9 to 10 days post-inoculation (dpi) (Figure 1A,B, “Symptoms”). Following this initial expression of disease symptoms, newly emerged leaves show a reduction in symptom severity (Figure 1C, “Pre-Recovery”). By 20 dpi, the third set of emerging leaves is nearly symptomless (Figure 1D, “Recovery”). This process has been termed symptom remission or recovery [12,13]. Similar observations with several plant viruses were reported as early as 1928 [14] and more recently, in plants infected with geminiviruses [15-17]. In PepGMV-infected pepper plants, the concentrations of viral ssDNA and viral mRNA in recovered leaves are dramatically reduced compared to the viral nucleic acid concentrations in severely symptomatic leaves [12,13]. Based on the presence and characterization of small RNA of viral origin (svRNA), as well as the levels of methylated viral DNA by host methylation machinery, the recovery process has been associated with transcriptional and post-transcriptional gene silencing mechanisms [13]. However, little is known in the pepper-PepGMV recovery system with respect to changes in host gene expression that occur during infection.

For organisms with no publicly available genome sequence, such as pepper, transcriptome analyses can provide major insights into genes involved in important biological processes. Deep transcriptome sequencing technologies, such as pyrosequencing provided through the Roche/454 sequencing platform [18], is a powerful tool for the identification of transcripts and transcript variation in plant-pathogen interactions [19-21]. In this study, transcriptional variation in pepper during PepGMV infection was analyzed in healthy (mock), symptomatic, and recovered pepper leaves. To provide insight into the geminivirus-host molecular interactions in the pepper-PepGMV recovery system, we employed next generation transcriptome sequencing of viral-infected pepper leaves and through differential gene expression, identified key transcripts (genes) involved in this phenomenon. We report a total of 309 differentially expressed (DE) genes in the pepper-PepGMV recovery system with major differences in up- and down-regulated genes observed between healthy (mock) and symptomatic or recovered tissues. Of these, 246 have a known function including genes that are associated with defense responses such as pathogenesis-related (PR) proteins, reactive oxygen species, jasmonic acid, and ethylene signaling pathways. A set of genes with a novel role in defense responses were also identified thereby expanding our understanding of the molecular interactions that underlie the PepGMV-pepper recovery system.

## Results & discussion

### Sampling & transcriptome sequencing

Four-leaf stage Chili pepper plants (*C. annuum* L cv. Sonora Anaheim) were inoculated by bombardment with dimeric PepGMV clones (Figure 1A) [12,13]. Symptom appearance was observed at 9 dpi in newly developed leaves (Figure 1B, “Symptoms”) and a decreased severity of symptoms was observed at 15 dpi in the next set of newly emerged leaves (Figure 1C, “Pre-Recovery”). The third pair of new leaves after inoculation emerged at 20 dpi and was symptomless (or showed an important reduction in the severity of the symptoms) (Figure 1D, “Recovery”). Symptomatic and recovered leaf tissues were sampled at 9 and 20 dpi, respectively, RNA isolated, and cDNA libraries constructed. As a control, total RNA from a pool of tissues (9, 15, and 20 dpi) was isolated from mock-inoculated healthy pepper leaves and a single cDNA library was constructed.

All of the libraries were sequenced using pyrosequencing on the Roche 454 GS20 platform [18]. A total of 1,838,567 reads were obtained from nine pyrosequencing runs. The number of runs and reads for each condition were mock-inoculated (1 run, 222,558 reads), symptomatic tissue at 9 dpi (5 runs, 865,103 reads), and recovered tissue at 20 dpi (3 runs, 750,906 reads) (Table 1).

**Table 1:** Summary of nine 454-pyrosequencing runs used in this study

**Tissue**	**Number of runs**	**Total no. reads**	**Average length (nt)**	**Average quality**
**Mock**^a^	1	222,558	99.4	27.8
**Symptomatic**^b^	5	865,103	98.6	28.1
**Recovered**^c^	3	750,906	101.7	27.5
**Total**	**9**	**1,838,567**	99.8	

^a^ Pool of mock-inoculated leaf tissue sampled at 9, 15 and 20 dpi.

^b^ Pool of symptomatic leaf tissue sampled at 9 dpi. A single run had low quality sequences and thus was discarded from downstream analyses. A total of 770,694 sequences from a total of 4 runs were used in downstream analyses of symptomatic tissues.

^c^ Pool of recovered leaf tissue sampled at 20 dpi.

### Differential expression analyses

To measure transcript abundances for each condition (Mock (M), Symptomatic (S), and Recovered (R)), pyrosequencing reads were aligned using the BLASTN algorithm [22] to a *Capsicum annuum* Reference Transcriptome (CaRT) that contains 32,220 total transcripts representing 12.5 Mb [23]. Pyrosequencing reads were mapped against the CaRT dataset and alignments with an identity equal to or greater than 96.6% and alignment length equal to or greater than 30 bp were retained [24]. The number of pyrosequencing reads mapped to a specific CaRT transcript was used to estimate transcript levels in each condition (Mock, Symptomatic and Recovered) [24]. Read counts were normalized by using a relative frequency of reads, i.e., number of reads mapped for a given contig relative to the total number of reads for a specific library, a method used previously in efficient detection of DE genes [25].

To assess the technical reproducibility of the pyrosequencing runs, we compared different runs from a single condition (technical replicates) by calculating the transcript abundances from each independent run (*p* ≤ 2.26e-16) [24]. Correlation within the technical replicates from the recovered leaf library (e.g., R-run1 vs. R-run2; R-run2 vs. R-run3; R-run1 vs. R-run3) revealed no differential gene expression between the 454 runs indicating a high degree of technical replication (Additional file 1). Examination of the number of reads, average length, and average quality on the technical replicates of the symptomatic library revealed a single run with low quality; this single run was discarded leaving a total of 4 runs with a total of 770,694 reads from the symptomatic library that were used in downstream analyses. The high degree of technical replication between the different 454 runs of the symptomatic leaf library is shown in Additional file 2.

**Figure 2:**
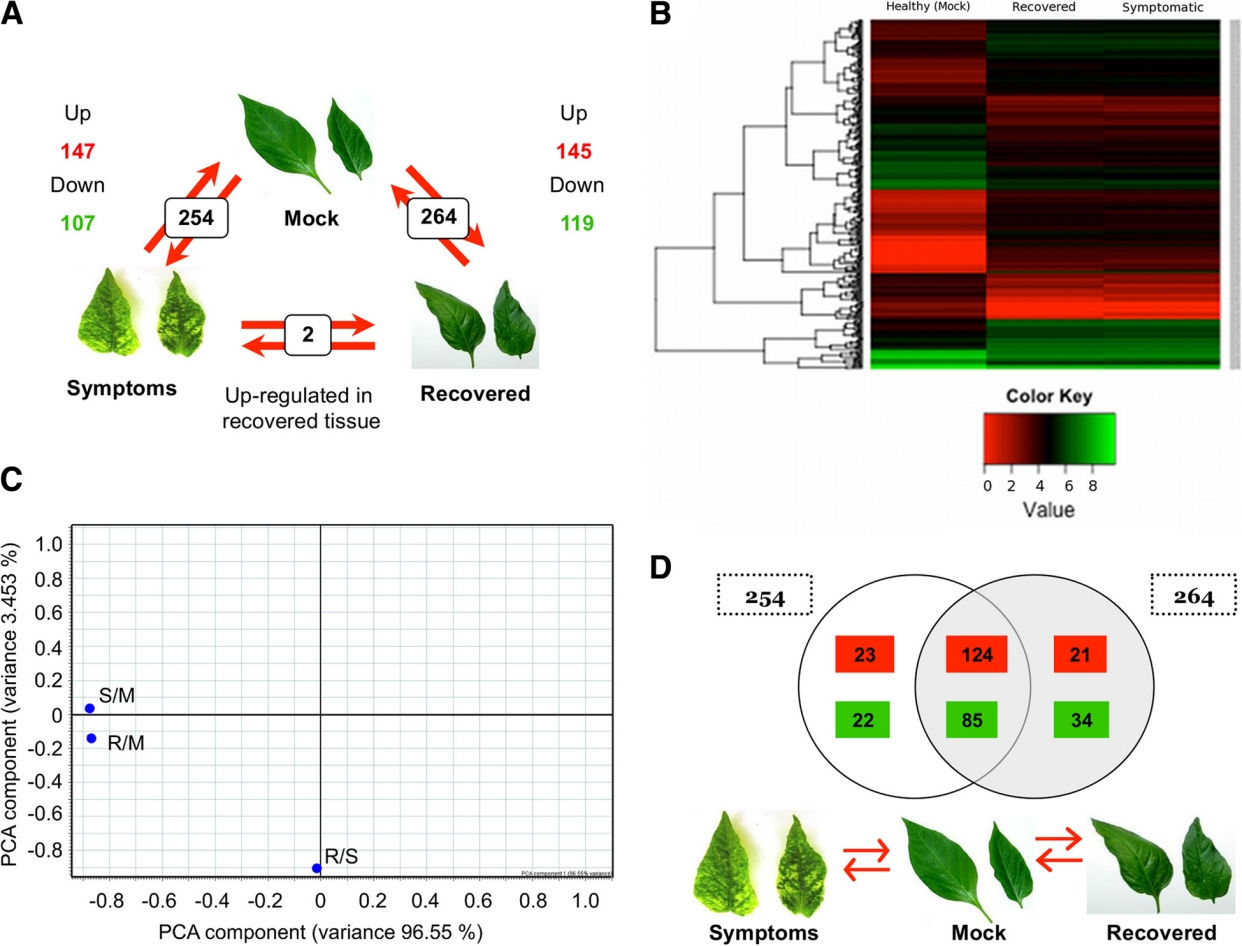
**Computationally predicted differentially expressed genes.A**) Symptomatic vs. mock comparison revealed a total of 254 differentially expressed genes, of which 147 were up-regulated and 107 down-regulated. A comparison of recovered vs. mock comparison revealed 264 differentially expressed genes, of which 145 were up-regulated and 119 were down-regulated. When symptomatic and recovered tissues were compared, only 2 genes were up-regulated in the recovered relative to symptomatic tissue. **B**) Hierarchical clustering of genes identified as differentially expressed in PepGMV-infected pepper plants. Log2 expression values were used for the analysis and negative values were set to zero. Clustering and the heat map were performed using R [71]. Green indicates down-regulated, red up-regulated and black unchanged values, as shown on the color scale at the side of the figure. **C**) Principal component analysis of differentially expressed genes. The percentage of variance explained by each component is shown within the brackets. R/S = Recovered vs. Symptomatic, S/M = Symptomatic vs. Mock and R/M = Recovered vs. Mock. **D**) Comparisons between S/M and R/M show that nearly 80% of differentially expressed genes are identical to both comparisons; 124 up-regulated (red) and 85 down-regulated (green) genes are differentially expressed in symptomatic and recovered tissues vs. mock-inoculated.

The Fisher’s exact test was used to estimate the probability of significant differences among the transcript abundances in pair-wise comparisons (Mock vs. Symptomatic; Mock vs. Recovered; Symptomatic vs. Recovered) and significant differences were estimated using *p* ≤ 1.5e-6 (α = 0.1; probability of Error Type I) and the Bonferroni correction for multiple testing (Additional file 3). Comparisons of the symptomatic and recovered tissue relative to control leaves (mock-inoculated), revealed a total of 309 differentially expressed (DE) genes with 254 and 264 DE genes in symptomatic and recovered leaves relative to mock-inoculated leaves, respectively (Figure 2A). In symptomatic tissue, 147 and 107 genes out of 254 were up- and down-regulated relative to mock-inoculated leaves, respectively; whereas the recovered leaves, of 264 genes, 145 were up-regulated and 119 were down-regulated relative to mock-inoculated leaves (Figure 2A). Two genes were found up-regulated in recovered tissue relative to symptomatic tissue; one encodes a phosphodiesterase and the second gene has no known function. Expression patterns of the 309 DE genes in mock-inoculated, symptomatic and recovered tissues (Figure 2B), as well as hierarchical clustering of fold expression of the 309 DE genes in symptomatic and recovered tissue vs. mock-inoculated tissue (S/M, R/M) and recovered vs. symptomatic (R/S), revealed that expression profiles in symptomatic and recovered tissues are similar to each other (Additional file 4). Principal Component Analysis (PCA), showed that comparisons of fold-change of the 309 DE genes between the two stages (R/S) were significantly different than comparisons of the two stages relative to mock-inoculated tissue (S/M, R/M) (Figure 2C).

A comparative analysis of DE genes (up- and down-regulated) in both comparisons, symptomatic vs. mock and recovered vs. mock, showed that nearly 80% of the genes were in common to both groups; 124 up-regulated genes and 85 down-regulated genes were DE in both symptomatic vs. mock-inoculated and recovered tissue vs. mock-inoculated (Figure 2D). Some genes were differentially expressed only in symptomatic or recovered vs. mock-inoculated. Of these, 23 were up-regulated and 22 were down-regulated in symptomatic tissue, and in the recovered tissue, 21 up- and 34 down-regulated genes were identified. However, less stringent parameters revealed that most of these genes were also differentially regulated in both conditions (data not shown). Thus, the Fisher’s exact test for sensitivity failed to detect these genes as DE and examination of the expression differences indicate they were near the cutoff for annotating genes as differentially expressed (data not shown). Due to restrictive criteria used to define differential expression in this study, these data should be considered an under-estimation of differential expression in the response of pepper to PepGMV.

### Functional annotation & gene ontology associations

A BLAST search (E ≤ 1e-06) with the 309 (168 up- and 141 down-regulated) DE genes revealed that 246 (79.6%) matched an entry in the National Center for Biotechnology Information (NCBI) non-redundant database. Gene Ontology (GO, [26,27]) associations were assigned by performing a BLASTX [22] search against the predicted *Arabidopsis thaliana* proteome [27,28] (E ≤ 1e-06) and transitively assigning GO terms for the biological process category from *A. thaliana* to the corresponding pepper genes. GO terms were further reduced to GO Slim terms by using GOTermMapper (http://go.princeton.edu/cgi-bin/GOTermMapper). Analysis of the DE genes revealed that 219 (70.8%) of the 309 transcripts had a significant alignment to the *A. thaliana* proteome. Of these, 136 were up-regulated and 83 were down-regulated. A total of 32 and 58 pepper up- and down-regulated genes, respectively, did not have a significant match to *A. thaliana* proteome and as a consequence, were not assigned a GO annotation.

**Figure 3:**
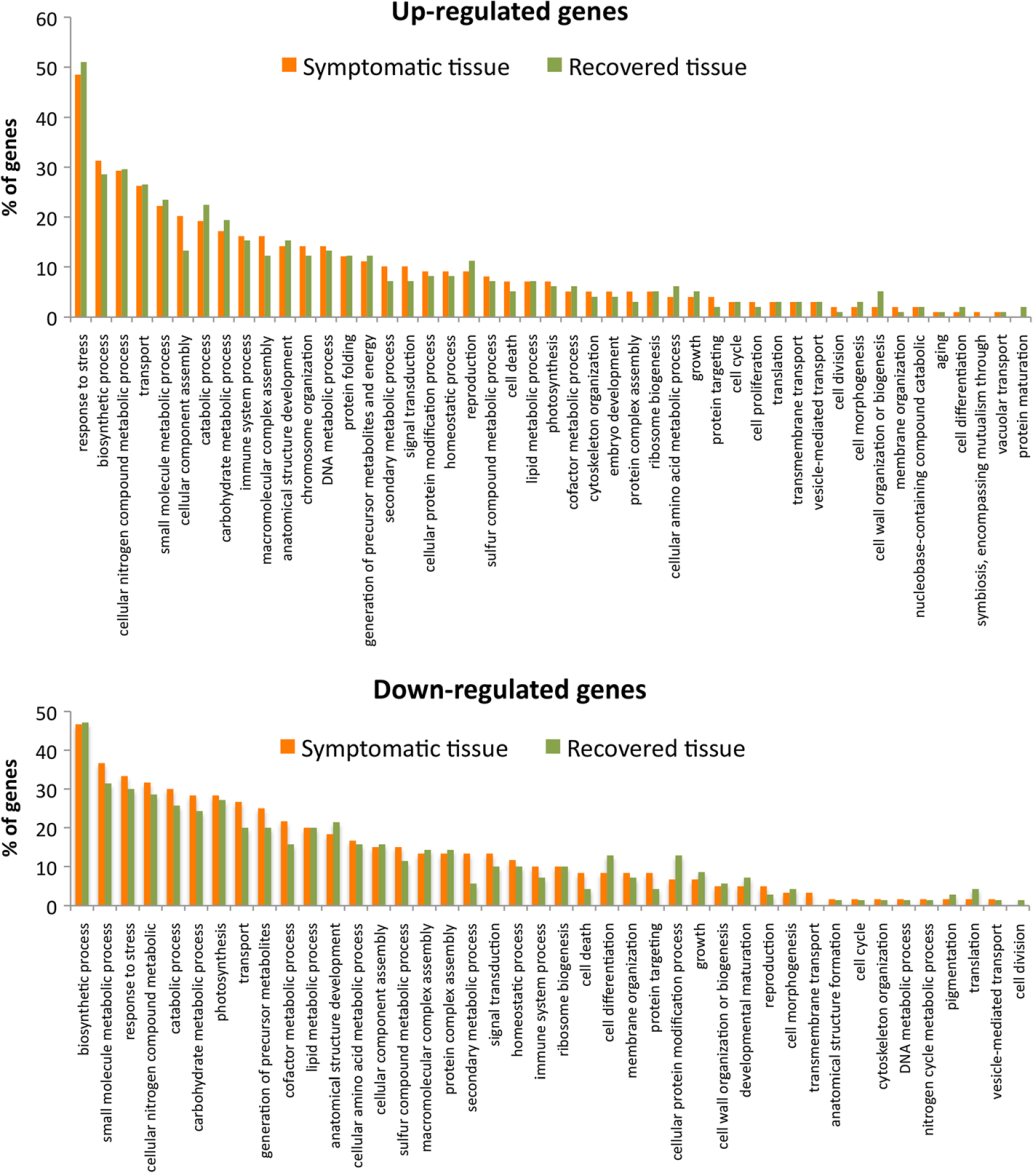
**Functional annotation of differentially expressed genes using Gene Ontology Slim terms **[26,27]. **A**) GO Slim terms for biological process assigned to up-regulated genes in symptomatic and recovered tissues relative to mock-inoculated tissues. **B**) GO Slim terms for biological process assigned to down-regulated genes in symptomatic and recovered tissues relative to mock-inoculated tissues. GO associations were assigned by a BLASTX [22] search against *A. thaliana* proteome and transitively assigning the GO term to the pepper transcript.

GO Slim terms associated with up-regulated transcripts (168 total genes) in symptomatic and recovered relative to the mock-inoculated tissues revealed 48 and 50 genes with GO associations classified as “responses to stress” in symptomatic and recovered tissues, respectively (Figure 3A). GO Slim terms assigned to down-regulated genes in symptomatic and recovered leaves (141 total genes) included 28 and 33 genes associated with “biosynthetic process”, respectively. In both recovered and symptomatic tissue, 18 down-regulated genes were associated with “catabolic process” and 18 down-regulated genes were associated with “photosynthesis” (Figure 3B). The GO results are consistent with the hypothesis that biotic stress marks a transition from growth and reproduction to physiology and metabolism tailored for defense responses [29] including a programmed down-regulation of primary metabolism.

### Signaling and pathogen response genes

To further characterize the PepGMV-pepper recovery process or phenomenon, genes encoding proteins implicated in defense functions, such as PR proteins, reactive oxygen species, jasmonic acid biosynthesis, and ethylene signal transduction were examined in depth in our set of differentially expressed genes. Although not all of the components for each of defensive pathway or response were present in our datasets, several well-known markers for these pathways were identified suggesting that these defense responses are activated in the pepper-PepGMV interaction. Transcripts encoding the pathogenesis-related proteins, *PR5* (Pepper00302) and two members of *PR1* gene family (Pepper27140, Pepper31625), were highly represented during PepGMV infection (Table 2). Genes involved in jasmonic and ethylene-mediated defense responses, *b-CHI* (basic chitinase; Pepper32368) and *HEL* (hevein-like; Pepper32274), were up-regulated in infected leaves. Interestingly, the mRNA encoding the final step of ethylene biosynthesis [30], *ACC* oxidase (Pepper01276), was up-regulated in both conditions (Table 2). In contrast, the *EIN3* (ethylene insensitive 3; Pepper00009) transcription factor, which is part of the ethylene signaling pathway [30] was down-regulated in infected tissues (Table 2). The transcripts for components of the jasmonic acid pathway, *OPR1* (12-oxophytodienaote reductase 1; Pepper26071) and *LOX1* (lipoxygenase; Pepper31749), were elevated in both conditions (Table 2).

**Table 2:** Differential expression of genes involved in the oxidative response, pathogenesis, jasmonic acid biosynthesis and ethylene responses during the pepper-PepGMV interaction

**ID**	**GENE NAME**	**Fold-change**		
	**Oxidative response**	**R / S**	**S / M**	**R / M**
Pepper28784	CAT	0.94	3.67	3.48
Pepper25924	ANN4	1.07	7.42	7.98
Pepper30410	ANN1	1.11	4.26	4.74
Pepper28222	GST1	1.08	4.82	5.24
	**Pathogenesis-related protein**			
Pepper00302	PR-5	0.97	25.05	24.37
Pepper27140	PR-1	1.25	14.22	17.89
Pepper31625	PR-1	1.11	16.08	17.89
	**Ethylene and jasmonic acid**			
Pepper01276	ACC	0.98	2.02	1.99
Pepper00009	EIN3	1.18	0.48	0.57
Pepper26071	OPR1	0.90	2.76	2.51
Pepper31749	LOX1	0.99	3.48	3.47
Pepper32274	HEL	1.08	2.25	2.43
Pepper32368	b-Chi	1.04	39.59	41.35
	**Others**			
Pepper05849	RRP1	0.96	69.59	66.96

Transcripts involved in oxidative stress and redox signaling were up-regulated in both conditions relative to mock-inoculated tissue including *CAT* (catalase; Pepper28784) which is involved in scavenging H_2_O_2_[31], consistent with results reported by [32] in which *CAT* is induced in PepGMV-infected *Capsicum chinense* plants. Recent data suggests that annexins may play an important role in conferring oxidative protection [33] and elevated levels of mRNAs that encode for annexin (*ANN*) (Pepper25924 and Pepper30410) were observed during PepGMV infection. *GST1* (glutathione S-transferase) is hypothesized to enhance oxidative stress tolerance [34] and is induced by salicylic acid, jasmonic acid, ethylene, and hydrogen peroxide [35]. In this study, *GST1* (Pepper28222) was induced in both symptomatic and recovered tissues relative to mock-inoculated tissues (Table 2), consistent with previous studies with common bean (*Phaseolus vulgaris*) and the geminivirus *Bean dwarf mosaic virus* (BDMV) [36].

One of the most up-regulated genes in this study encodes a kiwelling ripening-related protein 1 (Pepper05849; from here on referred to as RRP1). The mRNA for this gene was up-regulated 69.5 and 66.9 fold in symptomatic and recovered tissues relative to mock-inoculated tissues, respectively. RRP1 has not been previously reported as related as a plant defense-related gene, although, based on its characterized biochemical features, it may be a new type of PR protein [37].

Overall, these results are consistent with observations reported by [38] in which expression profiles of *A. thaliana* infected with the geminivirus *Cabbage leaf curl virus* (CaLCuV) were analyzed utilizing microarrays. The suite of genes identified in CaLCuV-infected *A. thaliana* leaves mirrored the DE genes identified in the pepper-PepGMV recovery system in this study [38]. One exception is *EIN3, which* was down-regulated at 9 and 20 dpi in the pepper-PepGMV recovery system (Table 2) yet in the CaLCuV-Arabidopsis interaction the Arabidopsis ortholog was up-regulated at 12 dpi [38] suggesting differences in the response of these two dicotyledonous plants to viral infection with respect to the ethylene signaling pathway.

### Experimental validation of differentially expressed genes by quantitative real-time PCR

**Figure 4:**
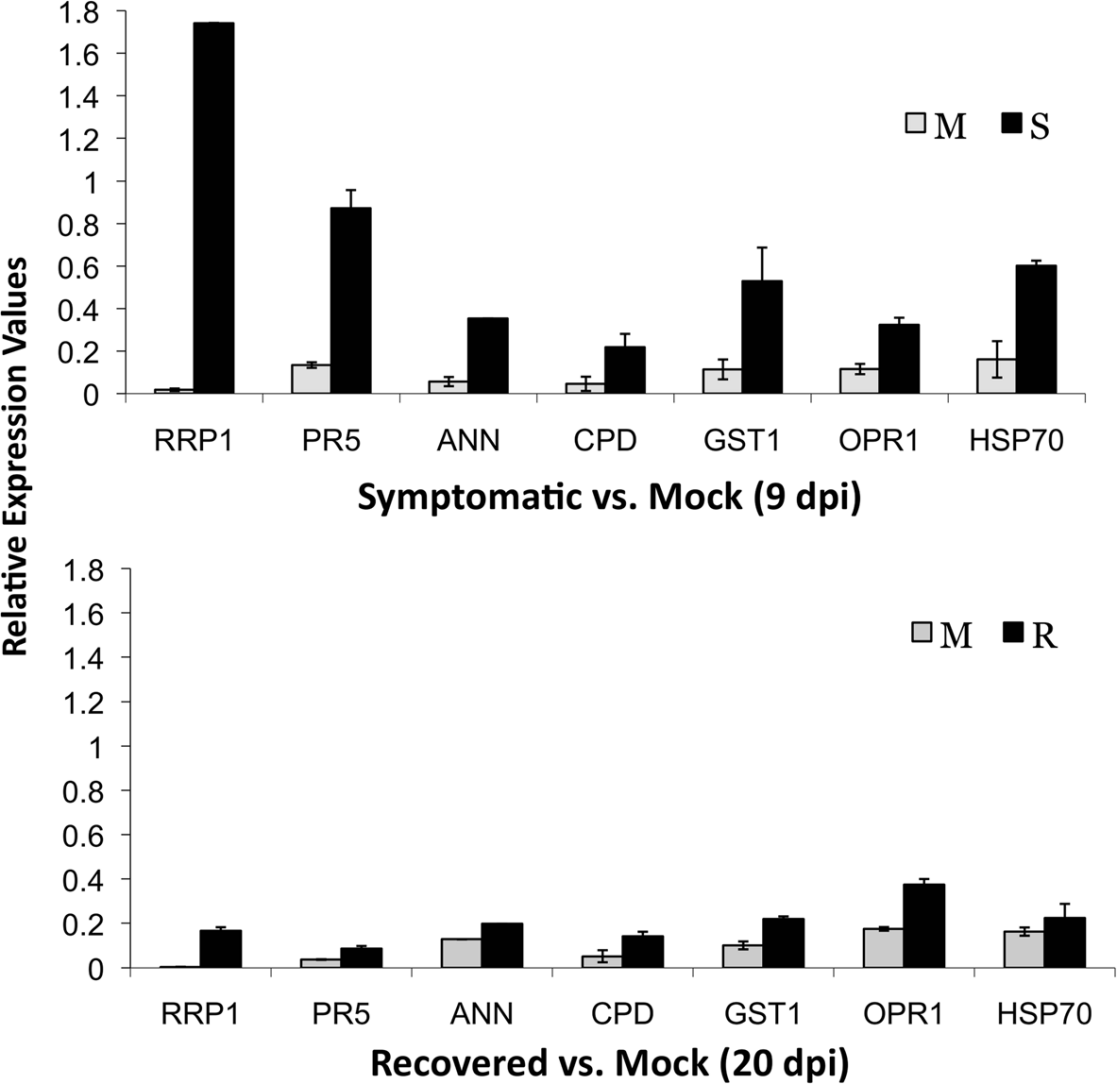
**Validation of computationally predicted differentially expressed genes in PepGMV-infected pepper plants.** Relative level of expression measured by quantitative reverse-transcription PCR. Error bars indicate standard deviation of mean for triplicate samples. Expression of β-tubulin was used for data normalization. *RRP1* (Kiwellin ripening-related protein); PR5 (thaumatin-like protein); *ANN* (annexin protein); *CPD* (carboxypeptidase protein); *GST1* (glutathione transferase); *OPR1* (12-oxophytodienoate reductase 1); *HSP70* (heat shock protein) M: mock; S: symptomatic; R: recovered.

Computationally determined differential expression was validated using quantitative reverse-transcription PCR assays (qRT-PCR). For this purpose, seven up-regulated genes from the Mock vs. Symptomatic and Mock vs. Recovered tissue comparisons were selected for qRT-PCR validation. These genes spanned a number of metabolic pathways as well as levels of computationally predicted expression levels (high, medium, and low; Table 3). As shown in Figure 4, the qRT-PCR assay confirmed the up-regulation of the seven selected genes computationally-predicted to be differentially expressed. An interesting difference, however, between the qRT-PCR results and the computationally-predicted results was noticed in the recovered tissue. In the qRT-PCR, the RNA levels detected for the seven genes analyzed were substantially reduced in the recovered tissue. On the other hand, the expression levels observed in the computationally-prediction analysis were basically similar in both symptomatic and recovered tissues.

**Table 3:** Computationally predicted differentially expressed genes

**Contig ID**	**Gen ID**	**Non-redundant polypeptide (NCBI)**	**Biological process (GO)**	**Symptomatic vs. Mock**				**Recovered vs. Mock**			
				**Mapped reads**		**Predicted fold change**	**qRT- PCR fold change**	**Mapped reads**		**Predicted fold change**	**qRT- PCR fold change**
				**S**	**M**			**R**	**M**		
Pepper05849(839 bp)	Pepper-RRP1	Putative kiwellin ripening-related protein precursor [S. tuberosum]	Gene Ontology annotation was not found	225	1	69.5	138.6	217	1	66.9	38.4
Pepper00302(756 bp)	Pepper-PR5	PR5-like protein [C. annuum]	Response to salt stress and bacterium.	81	1	25	6.4	79	1	24.3	2.6
Pepper25924(1224 bp)	Pepper-ANN	PREDICTED: annexin D4 [Vitis vinifera]	Response to abscisic acid stimulus and osmotic stress	192	8	7.4	6.3	207	8	7.9	2.4
Pepper27731(1768 bp)	Pepper-CPD	Carboxypeptidase type III [Theobroma cacao]	Gene Ontology annotation was not found	150	7	6.6	6.7	143	7	6.3	2.7
Pepper28222(845 bp)	Pepper-GST1	Glutathione S-transferase (GST1) [C. chinense]	Defense response to bacterium, salt stress, defense, cold, toxin catabolic process	187	12	4.8	4.4	204	12	5.2	2.2
Pepper26071(1240 bp)	Pepper-OPR1	12-oxophytodienoate reductase 1 (OPDA-reductase 1) (LeOPR1)	Jasmonic acid biosynthetic process	170	19	2.7	3.4	155	19	2.5	2.1
Pepper31770(1251 bp)	Pepper-HSP70	Heat shock 70 kDa protein	Response to cadmium ion, protein folding, bacterium, heat, high light intensity, hydrogen peroxide and virus.	303	36	2.6	2.9	323	36	2.7	1.3

RRP1 (Pepper05849) was computationally predicted to be up-regulated 69.5-fold and 66.9-fold in symptomatic and recovered tissue, respectively, relative to mock-inoculated leaves. qRT-PCR showed up to 138.6-fold expression difference in symptomatic tissue and 38.3-fold in recovered tissue (Table 3). Computational predictions and qRT-PCR analysis indicated that this transcript is highly abundant in symptomatic leaves (9 dpi), however the abundance decreases in recovered tissue (20 dpi) (Table 3, Figure 4). The role of PR genes in plant defense has been widely documented [39-43] and *PR5* (thaumatin-like protein) has been used as a marker of systemic acquired resistance, SAR [42,44]. Our computational analyses showed that Pepper00302 which encodes a *PR5* was up-regulated 25-fold in symptomatic and 24.4-fold in recovered tissues. qRT-PCR results showed 6-fold and 2.3-fold up-regulation in symptomatic and recovered tissues, respectively, relative to mock-inoculated tissues (Figure 4, Table 3). Interestingly, similar results have been shown in the BG-3821 accession of *C. chinense* Jacq., that displays resistance to PepGMV infection [32].

Pepper25924, which encodes a predicted annexin (*ANN4*), was computationally predicted to be 7-fold up-regulated in symptomatic and recovered compared to mock-inoculated tissue. qRT-PCR assays showed 6.3-fold and 2.4-fold up-regulation in symptomatic and recovered tissues, respectively, compared to the mock-inoculated tissue (Figure 4, Table 3). Annexin genes have been reported to have peroxidase activity, and it has been hypothesized that annexins can sense reactive oxygen species and modulate endogenous reactive oxygen species responses [33]. Studies in the PepGMV-resistant accession (BG-3821-R) of *C. chinense* have shown that PepGMV infection is able to trigger a reactive oxygen species-mediated response [32]. Additionally, it is hypothesized that a failure to control reactive oxygen species can lead to cell death [42,45] as the cellular damage resulting from high reactive oxygen species levels show hallmarks of necrosis [45]. It is important to note that the recovery phenomenon in the pepper-PepGMV system does not involve programmed cell death or the hypersensitive response. Thus, the annexin peroxidase activity could enhance oxidative tolerance by regulating reactive oxygen species levels in the PepGMV-infected leaves thereby resulting in the absence of a hypersensitive phenotype.

The computational prediction for Pepper27731, which encodes a serine carboxypeptidase (*SCP*), is 6-fold up-regulated in symptomatic and recovered tissues relative to mock-inoculated tissues. qRT-PCR results showed this gene 6.7- and 2.7-times up-regulated in symptomatic and recovered tissues, respectively, relative to mock-inoculated leaves (Figure 4, Table 3). Serine carboxypeptidases have been identified in many plant species [46]. The physiological role of serine carboxypeptidases in plant defense is unclear; however, characterization of this protein in different plant-pathogen systems suggests that it is required for the synthesis of defense compounds [46,47].

Pepper28222 encodes a *GST1* (glutathione-S-transferase 1) which has been demonstrated to be transcriptionally activated by reactive oxygen species [41]. Computational predictions revealed that Pepper28222 was up-regulated 4.8-fold in symptomatic and 5.4-fold in recovered leaves relative to mock-inoculated leaves. qRT-PCR results were similar, 4.2- and 2.2-fold up-regulation in symptomatic and recovered tissues, respectively, relative to mock-inoculated leaves (Figure 4, Table 3). Studies in *A. thaliana* infected with *Cauliflower mosaic virus,* a DNA virus*,* have shown that the activity of this gene is associated with both local and systemic accumulation of H_2_O_2_[41].

Jasmonic acid is a key signal transducer in the production of phytoalexins [48], which are important compounds for plant defense. Pepper26071 encodes a 12-oxophytodienoic acid reductase (*OPR1*). OPR catalyzes the NADPH-dependent reduction of 12-oxophytodienoic acid (OPDA) into 3-oxo-2[(Z)-2’-pentyl]cyclopentane-1-octanoic acid (OPC-8:0), and this reaction is part of the biosynthetic pathway leading to jasmonic acid. Computationally, Pepper26071 was 2.7-fold up-regulated in symptomatic tissue and 2.5-fold up-regulated in recovered tissue relative to mock; qRT-PCR results showed this gene up-regulated 3.4-fold in symptomatic and 2.2-fold in recovered tissue relative to mock-inoculated leaves (Figure 4, Table 3).

Heat-shock proteins (*HSP*) are a central component of the cellular chaperone network and play a crucial role in maintaining protein homeostasis by re-establishing functional native conformations under environmental stress conditions [49]. Interestingly, yeast two-hybrid studies in the geminivirus-plant interaction with the bipartite *Abutilon mosaic virus* (AbMV) suggested that these proteins may interact with the viral movement protein, and therefore, have a role during geminiviral cell-to-cell transport [50]. Pepper31770, which encodes a HSP70 chaperone, was 2.6-fold up-regulated in symptomatic and 2.8-fold up-regulated in recovered tissues relative to the mock-inoculated leaves. qRT-PCR revealed 2.9- and 1.3-fold up-regulation in symptomatic and recovered tissues, respectively, relative to the mock-inoculated leaves (Figure 4, Table 3).

Overall, a good correlation (Pearson’s coefficient of determination; r^2^ = 0.7) between differential expression values obtained in our computational predictions and those obtained by qRT-PCR was observed (Additional file 5). Discrepancies between the methods to determine DE genes may be related to methodological differences including the normalization methods used in our RNA-seq dataset as it has been reported that different normalization procedures impact differential expression detection [51].

### Temporal expression patterns of differentially expressed genes during PepGMV infection

**Figure 5:**
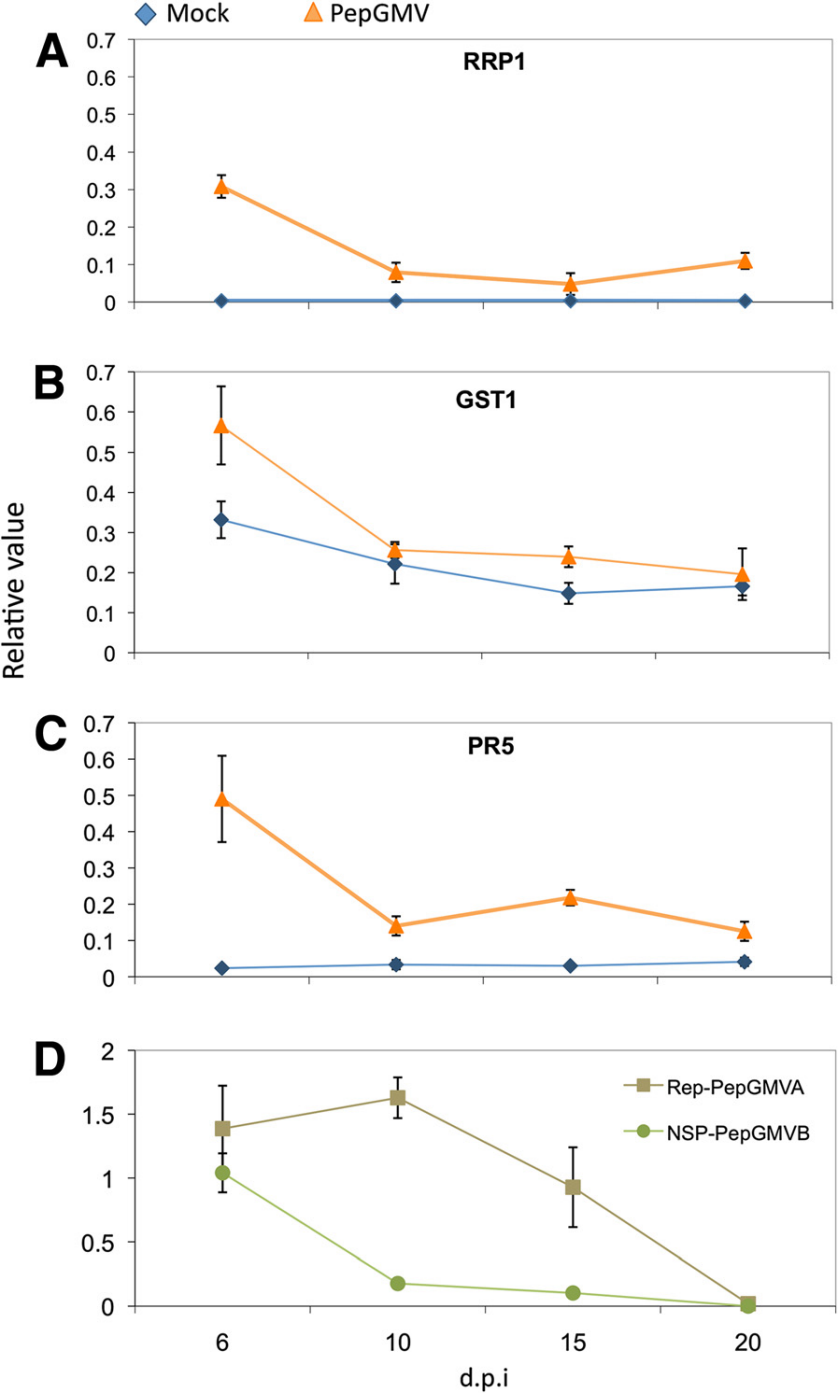
**Temporal gene expression patterns in the pepper-PepGMV recovery system.** Relative gene expression measured by quantitative reverse-transcription PCR at four time points: 6, 9, 15 and 20 days post-inoculation (dpi). Error bars indicate standard deviation of mean for triplicate samples and β-tubulin gene was used for data normalization. Mock: pepper plant mock-inoculated; PepGMV: pepper plants inoculated with PepGMV. **A**) *RRP1* (kiwellin ripening-related protein); **B**) *GST1* (glutathione S-transferase); **C**) *PR5* (thaumatin-like protein); **D**) Gene expression levels of *Pepper golden mosaic virus* (PepGMV), Rep (Replication-associated protein) and NSP (Nuclear shuttle protein).

The analysis of a subset of genes was further extended to a wider time course to determine the expression profiles during viral infection. Total RNA was isolated from pre-symptomatic (6 dpi), symptomatic (9 dpi), pre-recovery (15 dpi) and recovered (20 dpi) leaves (Figure 1) and pepper transcripts 05849, 00302 and 28222, encoding *RRP1*, *PR5*, and *GST1* genes, respectively, were examined for expression through the time course. qRT-PCR assay results showed that transcript levels of these genes increased significantly prior to the symptomatic stage (6 dpi) suggesting that a major differential transcriptional activity occurs in infected plants prior to symptom development (Figure 5A-C). As the PepGMV infection progressed, the levels of RRP1, GST1 and PR5 transcripts levels decreased (Figure 5A-C). The expression patterns of *RRP1*, *GST1* and *PR5* were correlated with levels of two PepGMV transcripts. Rep (Replication-associated protein) and NSP (Nuclear shuttle protein) from viral component A and B, respectively (Figure 5D). The results showed high levels of the Rep and NSP transcripts at 6 and 9 dpi, which gradually decreased during the recovery process (15–20 dpi). These results are consistent with previous studies [12,13,52] in which viral ssDNA and mRNA concentrations peaked at 9–10 dpi when symptoms develop in the first set of leaves following inoculation (Figure 1). By 20 dpi (typically the third set of leaves after inoculation), both viral DNA and RNA concentrations showed a reduction of 40 to 60% of the highest peak [12,13,52].

### Orthologous and paralogous clusters

**Figure 6:**
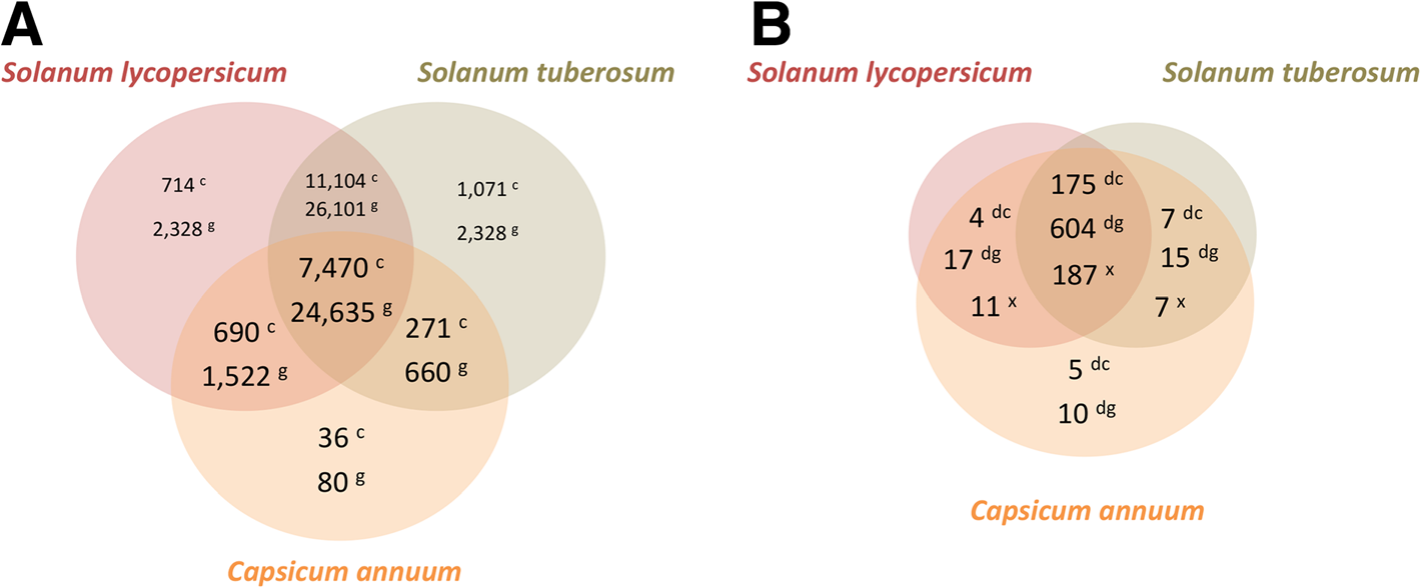
**Clusters of orthologous and paralogous genes families.** Predicted peptides from *Capsicum annuum*, *Solanum lycopersicum* and *Solanum tuberosum* were clustered using OrthoMCL [55]. **A**) Number of clusters (c) and genes (g) for each orthologous (*C. annuum*–*S. lycopersicum-S. tuberosum; C. annuum*–*S. lycopersicum*; *C. annuum–S. tuberosum*) and paralogous group within *C. annuum*. **B**) Numbers of differentially expressed genes (dg) grouped in orthologous clusters (dc) for the all three species, between two species (*C. annuum*–*S. lycopersicum*; *C. annuum–S. tuberosum*) and the paralogous group within *C. annuum.* The number of *C. annuum* genes (x) is represented in the intersections of the Venn diagram.

To determine if orthologs of our DE genes were present in the related *Solanaceae* crop species, tomato (*S. lycopersicum*) and potato (*S. tuberosum*), we generated orthologous clusters of the predicted proteomes of *C. annuum*[23], *S. lycopersicum*[53] and *S. tuberosum*[54] using the OrthoMCL algorithm [55] (Figure 6A). A total of 7,470 clusters containing 24,635 proteins were identified (Additional file 6) that represents the core proteome for all three species. Lineage-specific and two-species specific clusters were also identified. A total of 271 clusters containing 660 proteins were unique to *C. annuum* and *S. tuberosum* whereas 690 clusters with 1,522 proteins were unique between *C. annuum* and *S. lycopersicum* (Figure 6A). The OrthoMCL algorithm also identifies close paralogs within a species and we were able to identify 36 clusters grouping 80 proteins restricted to *C. annuum* (Figure 6A).

A total of 210 out of the 309 DE genes were identified in 191 clusters (Figure 6B; Additional file 6); of these, 187 of the DE genes were within 175 clusters that were shared by all three species (Figure 6B). Not all the transcripts could be clustered and 99 pepper transcripts remained as singleton transcripts (data not shown). A total of 11 DE pepper genes lacked a *S. tuberosum* ortholog and were restricted to a *C. annuumS. lycopersicum* orthologous group whereas seven DE pepper genes lacked a *S. lycopersicum* ortholog and were restricted to a *C. annuumS. tuberosum* orthologous cluster. The lineage-specific *C. annuum* DE genes could be clustered into 5 clusters containing 10 genes (Figure 6B). Pepper transcripts that encode proteins related to the oxidative response (*CAT, ANN4, ANN1* and *GST1*), pathogenesis-related protein 5 (*PR-5*), ethylene and jasmonic acid signaling (*ACC, EIN3, OPR1* and *LOX1*) and the novel gene *PRR1* were present within the three species orthologous clusters (Table 2; Additional file 6). Interestingly, transcripts encoding *HEL* (hevein-like protein) grouped in a lineage-specific cluster (Additional file 6). These data are consistent with previous reports that show a high degree of conservation within the Solanaceae family [56] and provide candidate genes for further investigation of the recovery process in solanaceous species.

### Identification of new components in the PepGMV-pepper recovery system

**Figure 7:**
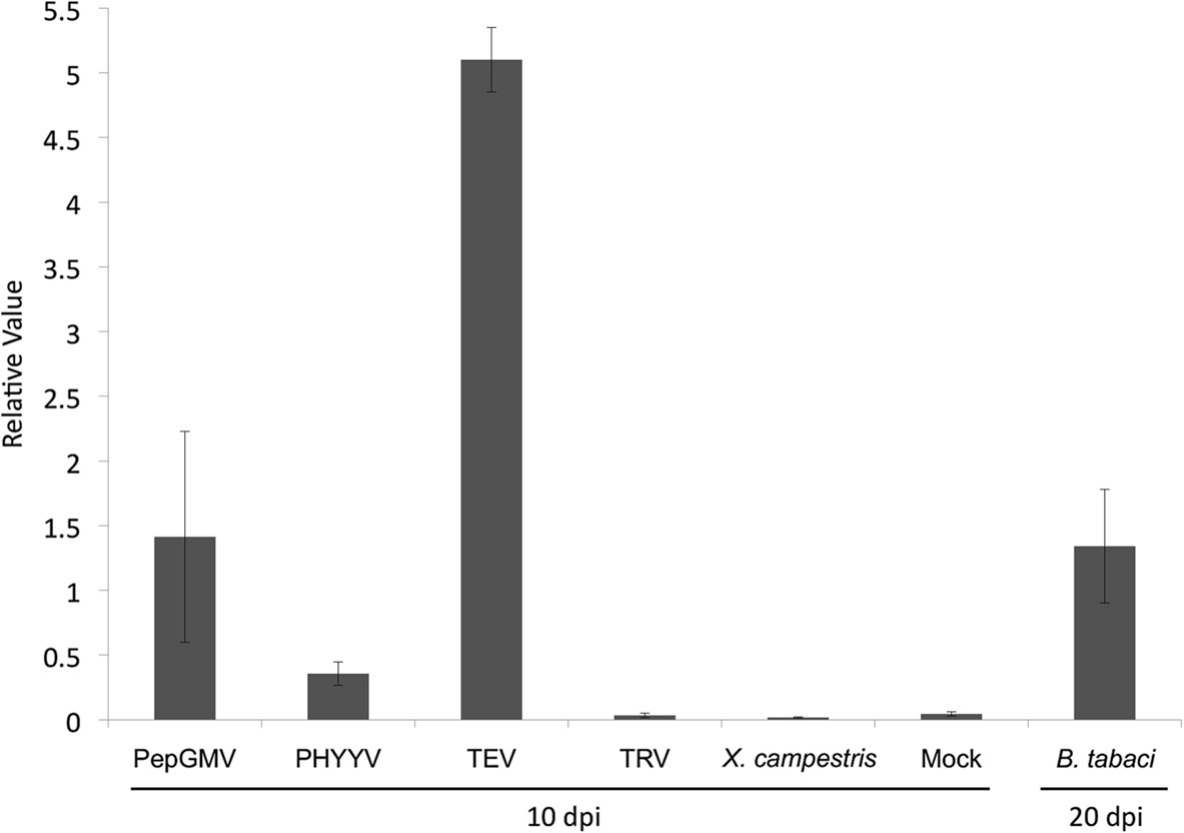
**Expression levels of the RRP1 (Ripening-related protein 1) gene under different biotic stresses.** Transcript abundance was measured by quantitative reverse-transcription PCR at 10 dpi in systemic pepper leaves infected by PepGMV (*Pepper golden mosaic virus*), PHYYV (*Pepper huasteco yellow vein virus*), TEV (*Tobacco etch virus*), *X. campestris* pv. *vesicatoria*, and infested with *B. tabaci* (whitefly). Error bars indicate standard deviation of mean for triplicate samples and β-tubulin gene was used for data normalization.

It has been reported that next generation sequencing methods are an excellent tool for the discovery of novel genes [19-21,57-59]. The results of this study suggest that the *RRP1* gene (Pepper05849) may have a role in plant defense. Interestingly, RRP1 is not expressed in mock-inoculated (healthy) plants but in PepGMV-inoculated plants, expression is high in pre-symptomatic, symptomatic and recovered leaves during the establishment of viral infection (Figure 5A). To assess whether the *RRP1* gene is exclusively expressed in response to viral infection, qRT-PCR assays were performed with pepper plants challenged with different pathogens. Pepper plants were infected with two DNA viruses (PepGMV and *Pepper huasteco yellow vein virus* (PHYYV)), two RNA viruses ((*Tobacco etch virus* (TEV) and *Tobacco rattle virus*, (TRV)), a bacterium (*Xanthomonas campestris* pv. *vesicatoria*), and infested with whiteflies (*Bemisia tabaci*), the insect vector for PepGMV (Figure 7). Total RNA from leaves infected with the different pathogens was isolated at 10 dpi and from leaves exposed to whiteflies for 20 days. Pepper-*RRP1* expression levels were measured in all samples. Infection with the DNA viruses (PepGMV and PHYVV) and TEV, as well as whitefly infestation, resulted in induction of *RRP1* expression (Figure 7). This result is consistent with transcriptome analysis of *A. thaliana* challenged with whiteflies in which genes involved in systemic acquired resistance, such as *PR1* and *PR5*, were induced [60]. The highest level of induction was observed in TEV-infected pepper plants suggesting that *RRP1* may have a generalized role in the response to a wide range of biotic stress defense mechanisms.

The core histones (H3, H4, H2A, H2B) are the components of the nucleosome complex, the basic unit of chromatin [61]. Geminiviral genomes are replicated and transcribed in infected plant cells through double-stranded DNA intermediates, which are then assembled into mini-chromosomes [3,4,62]. Recently, it has been shown that the host histone H3 interacts with both viral movement proteins, NSP and MP, creating a complex composed of H3, NSP, MP and viral DNA, suggesting that H3 may play a role in geminivirus cell-to-cell movement through the formation of a movement-competent complex [63]. Interestingly, genes encoding all four core histones were identified as differentially expressed during PepGMV infection. Pepper transcripts 01647, 30474, 31867 and 31973 are predicted to encode histone H3, whereas pepper contigs 26934, 28934, 30839, 31472 and 31544 are predicted to encode H4. Similarly, seven pepper contigs are predicted to encode histone H2A-related proteins (25732, 31056, 31911, 32303, 32359, 32453 and 32284) and three contigs were predicted to encode histone H2B (32370, 32371 and 32456). All of these contigs were up-regulated in symptomatic and recovered tissues (Additional file 3). In the OrthoMCL analysis with the 309 DE genes, one of the large gene families within the *C. annuum-S. lycopersicum-S. tuberosum* orthologous clusters contains 24 histone H4-encoding proteins, of these, three were from *C. annuum*. Two *C. annuum-S. lycopersicum-S. tuberosum* orthologous clusters contained histone H3 while histone H2A was contained within a single *C. annuum-S. lycopersicum-S. tuberosum* cluster. Interestingly, two genes encoding histone H4 protein were present within a *C. annuum*-specific paralogous cluster (Additional file 6). Detection of DE genes encoding core histones in our dataset suggests that these might have an important role in the response of pepper to virus, including the recovery process.

It has been also reported that host plants methylate viral chromatin as a defense against geminiviruses [13,64-66]. Thus, viral chromatin is a target for transcriptional gene silencing and post-transcriptional gene silencing [13] and previous studies have suggested that the geminivirus recovery system requires the host RNA-directed DNA methylation pathway [13,64]. Indeed, methylation-deficient mutants of *A. thaliana* are hyper-susceptible to geminivirus infection and histone H3 methylated at lysine 9 (H3K9) is highly represented in viral chromatin [64]. In addition, studies on chromatin structure and gene regulation have characterized a remodeling process called “histone replacement”, in which canonical histones (i.e., H2A) are substituted by histone variants [67]. One of the variants of H2A is H2A.Z and recently it was shown that histone H2A.Z may function to maintain the repressed or active transcriptional states of a number of genes related to the systemic acquired resistance response in *A. thaliana*[67].

Recent studies suggest that histone H2A.Z may be involved in the induction of *ERF1* (ETHYLENE RESPONSIVE FACTOR1) and *b-Chi*, genes that act downstream in the ethylene and jasmonic acid signaling pathways [61,68,69]. Pepper32368 encodes *b-Chi* and our computational predictions revealed that this gene is up-regulated 39.6 and 41.3 fold in symptomatic and recovered tissue, respectively (Additional file 3). Collectively, these results have yielded candidate genes to further investigate the potential role of host chromatin modification and post-transcriptional gene silencing in the pepper-PepGMV recovery system.

## Conclusions

Virus-induced gene expression in plants has been studied in many host-virus models, including several geminivirus [32,36,38]. In most cases, however, the models permit examination of changes between healthy or non-inoculated plants and the infected, symptomatic tissues [38]. The pepper-PepGMV recovery system affords the opportunity to examine susceptibility and recovery in the same system. Quantification and comparison of transcript abundances using deep transcriptome sequencing and the *C. annuum* reference transcriptome [23] allowed us to analyze the transcriptional status of PepGMV-infected plants during the initial symptom stage and subsequent recovered condition. Modification of transcript levels for many genes occurs prior to the appearance of symptoms with the highest peak of expressed genes observed around 6 dpi. Newly emerged leaves are nearly symptomless (recovery stage), thus analysis of DE genes suggests that several elements related to the defense machinery of the plant (PR proteins, reactive oxygen species, as well as jasmonic acid and ethylene signaling pathways) may contribute in pepper-PepGMV recovery system along with the previously reported PTGS and TGS mechanisms. Interestingly, novel genes, such as Pepper-RRP1 and histone proteins, were identified which may have a role in plant defense. The results presented in this study provide valuable information for our understanding of the underlying molecular mechanisms by which PepGMV-infected pepper plants recover from geminiviral infection.

## Material & methods

### Biological material & sampling

*C. annuum* L cv. Sonora Anaheim seeds, a susceptible cultivar [10,12], were germinated in plant growth chambers under an 18 hr light/6 hr dark photoperiod at 26 to 28°C for two weeks. At the four true leaf stage, plants were inoculated with PepGMV (A + B) dimeric clones using biolistics as previously reported [12]. Mock-inoculated plants were bombarded with gold particles alone. Leaf tissues were sampled at 6, 9, 15 and 20 dpi, frozen immediately in liquid nitrogen, and stored at −80°C until use.

#### Inoculation of PHYVV and TEV

Pepper plants at the four true leaf stage were inoculated with dimeric clones using biolistics as previously reported [12]. Leaf tissue was sampled at 6 and 10 dpi, frozen immediately in liquid nitrogen, and processed. TEV was inoculated using sap from a infected tobacco plant and carborundum.

#### Agroinoculation of TRV vectors

*Agrobacterium tumefaciens* carrying the TRV vector was grown in LB medium containing kanamycin 50 mg/L (Bristol-Myers Squibb de México), carbenicillin 100 mg/L (Pfizer, New York, NY, USA) and rifampicin 50 mg/L (Bristol-Myers Squibb de México). The bacterial pellet was diluted in buffer (10 mM MgCl_2_, 10 mM MES pH 5.6, 150 μM acetosyringone) to achieve an optical density of 1. The bacterial suspension was infiltrated into the main vein of pepper leaves using a 1 cc syringe. Leaf tissues were sampled at 6 and 10 dpi, frozen immediately in liquid nitrogen, and processed.

#### Inoculation of *X. campestris*

*X. campestris* pv. *vesicatoria* was grown for 48 hrs in 5 ml of NYB medium (Casein Peptone 10 g/L, yeast extract 5 g/L, NaCl 5 g/L pH 7) at 27°C. The bacterial suspension was infiltrated into the main vein of pepper leaves using a 1 cc syringe. Inoculated plants were incubated at 27°C and leaf tissues were sampled at 6 and 10 dpi, frozen in liquid nitrogen, and processed.

#### Infestation with *B. tabaci* (whitefly)

Pepper plants at the four true leaf stage were exposed to a colony of *B. tabaci* (approximately 30 whiteflies). Leaf tissues were sampled at 20 days post exposure, frozen in liquid nitrogen, and processed.

### RNA extraction & cDNA library preparation

Total RNA was extracted from frozen tissue using the combined method of Trizol and PureLink Micro-to-Midi Total RNA Purification System kit (Invitrogen, Carlsbad, CA). RNA purity was checked using the Agilent 2100 Bioanalyzer RNA 6000 Nano Assay chip (Agilent Technologies, Stockport, U.K). cDNA synthesis was performed from 3.5 μg of total RNA using the Message Amp-II kit (Ambion, Foster City, CA) following the manufacturer’s protocol as described earlier [70]. For RRP1 expression, total RNA was extract from frozen tissues using Trizol (Invitrogen, Carlsbad, CA, U.S.A.).

### GS20-454 sequencing

cDNA samples were prepared for GS20-454 pyrosequencing as described previously [18,70]. Nine runs of three cDNA libraries were performed resulting in 1,838,567 total reads. Sequences of the 454-G20 reads are publicly available for download from http://www.bioingenios.ira.cinvestav.mx:81/Joomla/ and in NCBI Sequence Read Archive (accession number SRA052606).

### *In silico* differential expression analysis

Stand-alone BLAST software [22] was obtained from NCBI (http://www.ncbi.nih.gov). The 454-pyrosequencing reads were aligned using BLAST to the *C. annuum* Reference Transcriptome [23]. A custom local MySQL database was constructed to store and query information from BLAST alignments. Using criteria described in [24], an alignment was considered significant if ≥ 30 bp aligned at ≥ 96.6% identity. One impact on differential expression detection is the normalization method, e.g., FPKM values (fragments per kilobase of exon model per million mapped reads). FPKM values are heavily affected by a relatively small proportion of highly-expressed genes and, as such, can introduce biased estimates of differential expression if these genes are differentially expressed across the conditions under comparison [51]. Therefore, we elected to normalize transcript levels using a relative frequency of reads, i.e., number of reads mapped for a given contig relative to the total number of reads for a specific library. This method has proven to be efficient in the identification of DE genes [25]. The fold change of DE genes was estimated by obtaining the ratio between the relative frequencies for the two conditions. The probability (*p*-value) and significant differences between the samples (symptomatic vs. mock; recovered vs. mock; symptomatic vs. recovered) were estimated using the Fisher’s exact test. To reduce false positives, the QVALUE software was used to adjust p-values obtained from the Fisher’s exact test [48]. Changes in signal intensity of ± 1.45 or higher/lower between treatments were considered highly significant (p-value 1.5e-6; 90% confidence). However, we focused on DE genes using traditional criteria from microarray experiments in which the cut-off threshold for up-regulated is ≥ 2 and down-regulated genes is ≤ 0.5.

### Functional annotation and OrthoMCL analysis

Transctripts were annotated using BLASTX [22] searches against non-redundant polypeptides database from NCBI and the *A. thaliana* proteome (TAIR10; arabidopsis.org) as described previously [23]. GO associations [26,27] were made by GOTermMapper (http://go.princeton.edu/cgi-bin/GOTermMapper).

Orthologs and close paralogs were identified in the three predicted proteomes using OrthoMCL (v 1.4) [55] using the default parameters with an E-value cutoff of 1e-10. Transposable elements were filtered out to avoid clusters comprised entirely of transposable elements.

### Evaluation of genes expression by quantitative reverse-transcription PCR (qRT-PCR)

The same total RNA samples used for 454-pyrosequencing sequencing were used in the qRT-PCR validation experiments. Biological replicates confirmed the qRT-PCR results. DNA contamination was determined by running a PCR under the same conditions for the RNA samples. DNA-free RNA (1 μg) was used for cDNA synthesis using Superscript II Reverse Transcriptase (Invitrogen, Carlsbad, CA). qRT-PCR was carried out as previously described [12]. The primers used in this study are described in Additional file 7.

## Abbreviations

CaRT: *Capsicum annuum* Reference Transcriptome; M: Mock-inoculated tissue; PepGMV: *Pepper golden mosaic virus*; PHYVV: *Pepper huasteco yellow vein virus*; PTGS: Post-transcriptional gene silencing; qRT-PCR: Quantitative reverse transcription PCR; R: Recovered tissue; ROS: Reactive oxygen species; S: Symptomatic tissue; ssDNA: Single stranded DNA; svRNA: Small RNA of viral origin; TEV: *Tobacco etch virus*; TGS: Transcriptional gene silencing; TRV: *Tobacco rattle virus*.

## Competing interests

The authors declare that they have no competing interests.

## Authors’ contribution

EGC designed methods and experiments; performed the plant inoculations, RNA isolations, qRT-PCR assays, and bioinformatics analysis; analyzed and interpreted the data; and wrote the manuscript. EIL designed methods and experiments, constructed the cDNA library for pyrosequencing, performed the statistical analysis and collaborated to draft the manuscript. DTS designed methods and experiments, carried out plant inoculations, RNA isolation and qRT-PCR assays. RRB coordinated the project, designed experiments and collaborated in analyzing data and writing the manuscript. All the authors have read and approved the final manuscript.

## Supplementary Material

Additional file 1 **Correlation of aligned 454-reads to the *Capsicum annuum *Reference Transcriptome (CaRT) between different 454-runs from the recovered leaf cDNA library.** The best fitting linear correlations for each pair-wise comparison was calculated (p < 2.26e-16): run1 vs. run2 (r^2^ = 0.9666), run1 vs. run3 (r^2^ = 0.9698) and run2 vs. run3 (r^2^ = 0.9703). Click here for file

Additional file 2 **Correlation of aligned 454-reads to the *Capsicum annuum *Reference Transcriptome (CaRT) between different runs derived from the symptomatic leaf cDNA library.** The best fitting linear correlations for each pair-wise comparison was calculated (p < 2.2e-16): run1 vs. run2 (r^2^ = 0.9657), run1 vs. run3 (r^2^ = 0.9644), run1 vs. run4 (r2 = 0.9705), run2 vs. run 3 (r^2^ = 0.9658), run2 vs. run5 (r^2^ = 0.9667), run3 vs. run5 (r^2^ = 0.9663). Low quality was observed on run4 and therefore it was discarded from the downstream analysis (data not shown). Click here for file

Additional file 3 Table of differentially expressed genes with Fisher’s exact test p-values and fold-changes of pair-wise comparisons. Click here for file

Additional file 4 **Hierarchical clustering of differentially expressed genes identified in PepGMV-infected pepper plants.** A total of 309 genes were identified (fold change of at least ± 2 and p-value ≤ 1.57e-06) and the ratio for each comparison (Symptomatic (S) vs. Mock (M); Recovered (R) vs. Mock; Recovered vs. Symptomatic) was used for the analysis. Clustering was performed using the Smooth correlation and average linkage clustering in GeneSpring GX 7.3.1 software (Agilent Technologies^®^). Green indicates down-regulated, red up-regulated and black unchanged values, as shown on the color scale at the side of the figure. Click here for file

Additional file 5 Pearson’s coefficient of determination was obtained by log2 transformation of the computational predictions and qRT-PCR expression values. Click here for file

Additional file 6 **Table S1. Clusters of orthologous and paralogous genes families in *S. lycopersicum, S. tuberosum *and *C. annuum *species.** Table S2. Clusters of orthologous and paralogous of differentially expressed genes in *S. lycopersicum, S. tuberosum* and *C. annuum* species. Predicted peptides from *S. lycopersicum, S. tuberosum* transcripts were clustered using OrthoMCL [55]. Click here for file

Additional file 7 List of primers used in qRT-PCR assays. Click here for file
