# Supplementary figures and images for: Transcriptome analysis of symptomatic and recovered leaves of geminivirus-infected pepper (Capsicum annuum)
Source: Virol J. 2012 Nov 27;9:295. doi: 10.1186/1743-422X-9-295 (PMC3546870; doi:10.1186/1743-422X-9-295)

# Recovered tissue

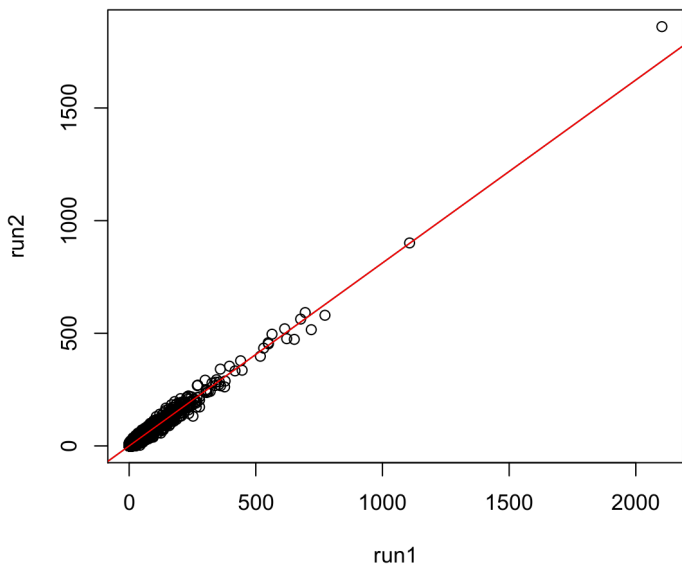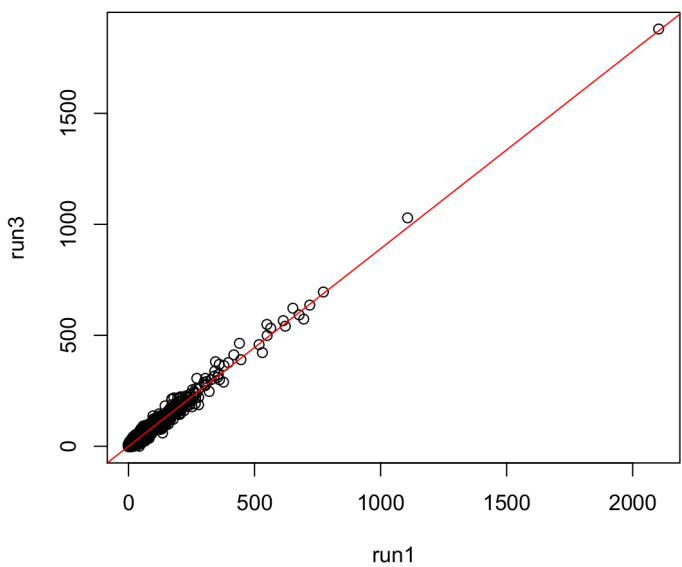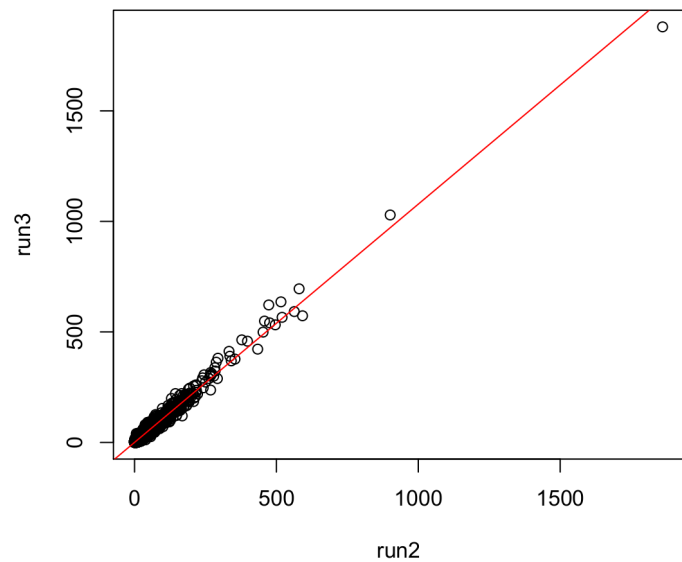

Supplement: Additional file 1 — Correlation of aligned 454-reads to the Capsicum annuum Reference Transcriptome (CaRT) between different 454-runs from the recovered leaf cDNA library. The best fitting linear correlations for each pair-wise comparison was calculated (p < 2.26e-16): run1 vs. run2 (r2 = 0.9666), run1 vs. run3 (r2 = 0.9698) and run2 vs. run3 (r2 = 0.9703). [file 1743-422X-9-295-S1.pdf]

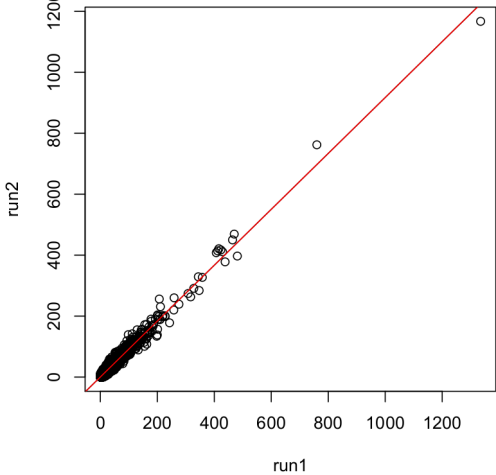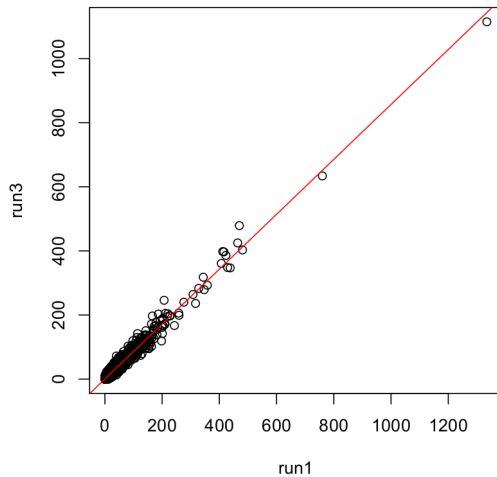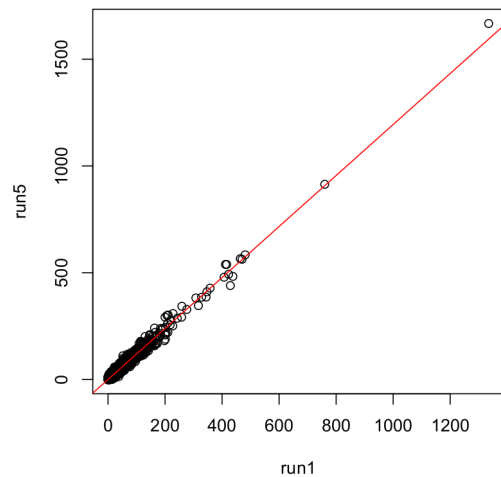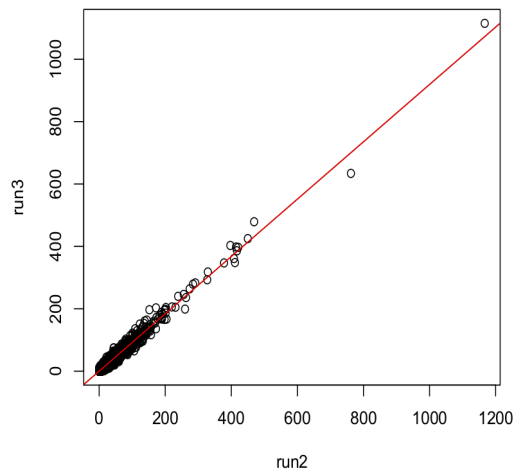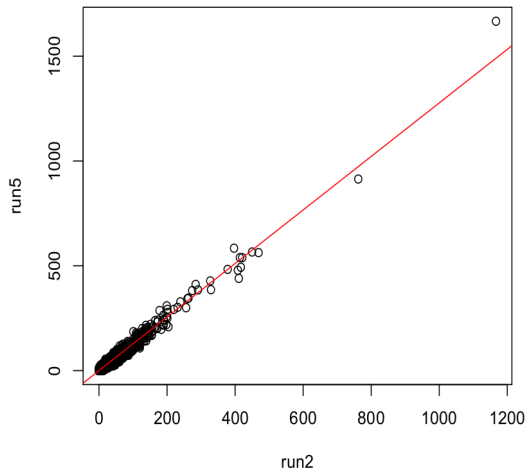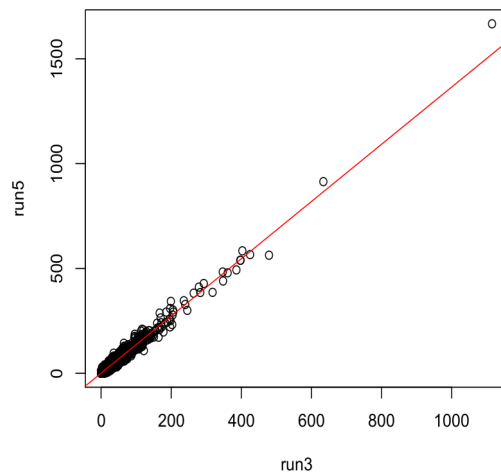

Supplement: Additional file 2 — Correlation of aligned 454-reads to the Capsicum annuum Reference Transcriptome (CaRT) between different runs derived from the symptomatic leaf cDNA library. The best fitting linear correlations for each pair-wise comparison was calculated (p < 2.2e-16): run1 vs. run2 (r2 = 0.9657), run1 vs. run3 (r2 = 0.9644), run1 vs. run4 (r2 = 0.9705), run2 vs. run 3 (r2 = 0.9658), run2 vs. run5 (r2 = 0.9667), run3 vs. run5 (r2 = 0.9663). Low quality was observed on run4 and therefore it was discarded from the downstream analysis (data not shown). [file 1743-422X-9-295-S2.pdf]

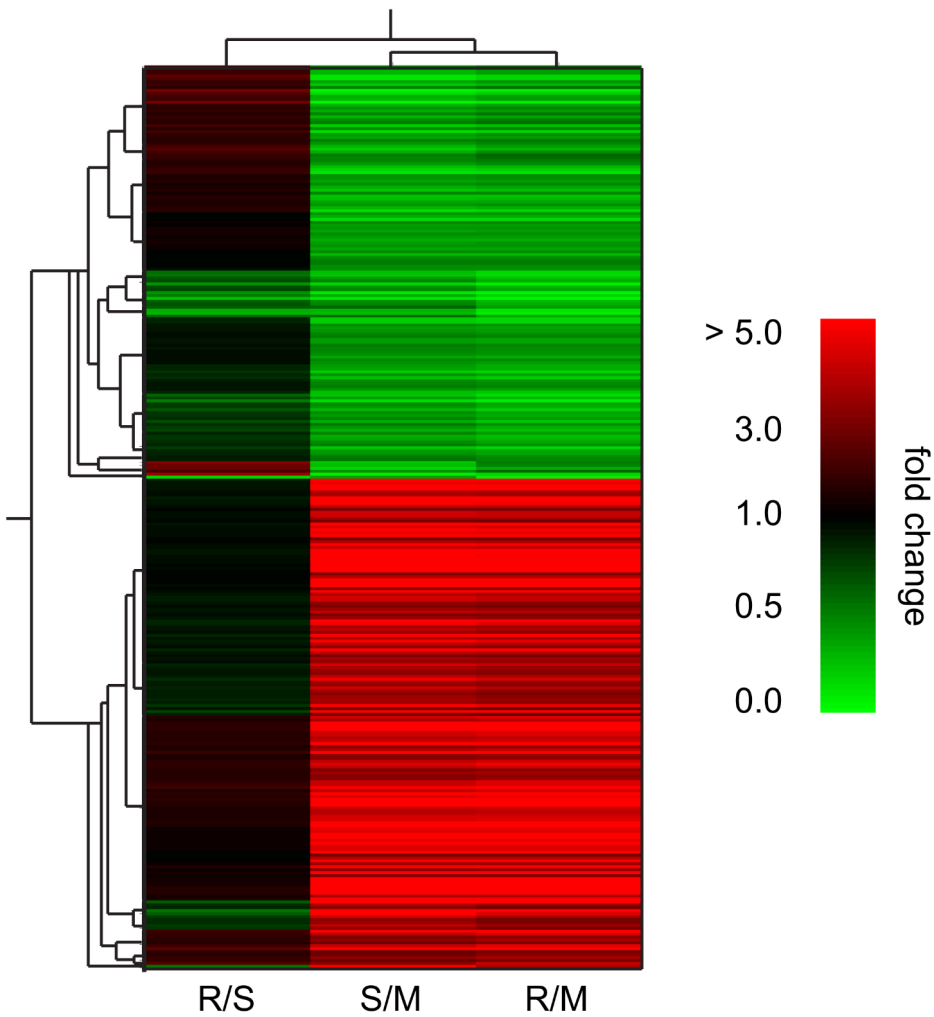

Supplement: Additional file 4 — Hierarchical clustering of differentially expressed genes identified in PepGMV-infected pepper plants. A total of 309 genes were identified (fold change of at least ± 2 and p-value ≤ 1.57e-06) and the ratio for each comparison (Symptomatic (S) vs. Mock (M); Recovered (R) vs. Mock; Recovered vs. Symptomatic) was used for the analysis. Clustering was performed using the Smooth correlation and average linkage clustering in GeneSpring GX 7.3.1 software (Agilent Technologies®). Green indicates down-regulated, red up-regulated and black unchanged values, as shown on the color scale at the side of the figure. [file 1743-422X-9-295-S4.pdf]

qRT-PCR

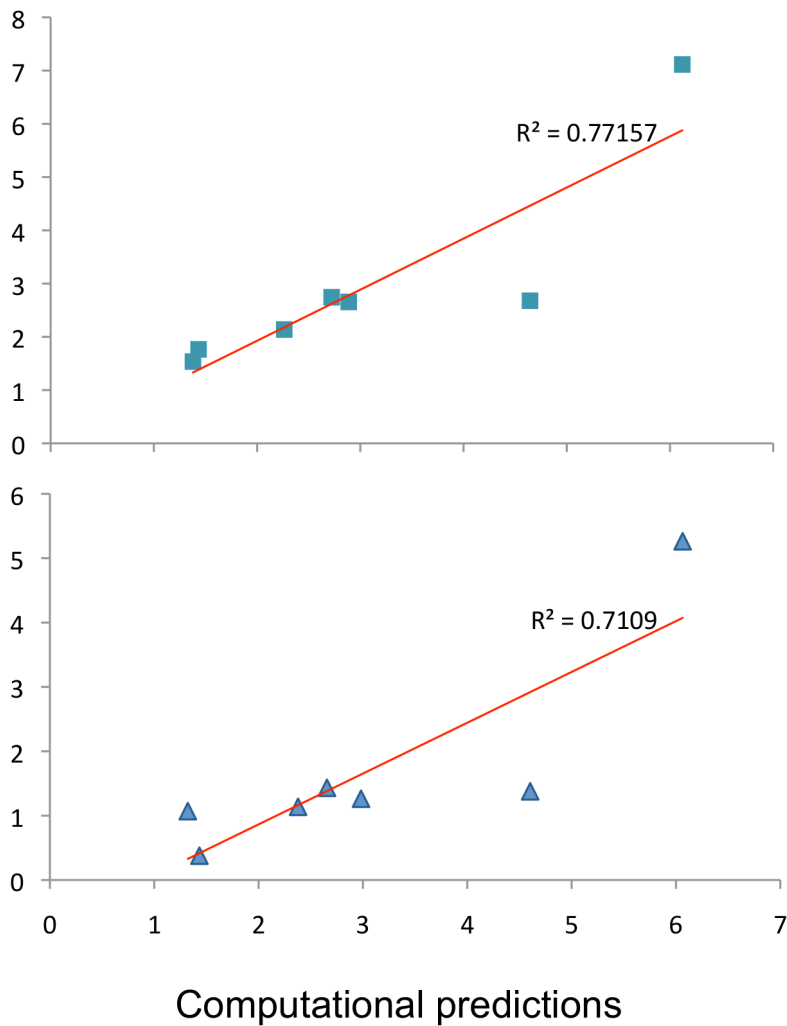

Supplement: Additional file 5 — Pearson’s coefficient of determination was obtained by log2 transformation of the computational predictions and qRT-PCR expression values. [file 1743-422X-9-295-S5.pdf]
